# The mutation landscape of *Daphnia obtusa* reveals evolutionary forces shaping genome stability

**DOI:** 10.1093/molbev/msag037

**Published:** 2026-02-09

**Authors:** Ruyun Deng, Feng Guo, Fei Fan, Wen Wei, Michael E Pfrender, Jeff L Dudycha, Michael Lynch, Zhiqiang Ye

**Affiliations:** Key Laboratory of Pesticide & Chemical Biology of Ministry of Education, School of Life Sciences, Central China Normal University, Wuhan 430079, China; Key Laboratory of Pesticide & Chemical Biology of Ministry of Education, School of Life Sciences, Central China Normal University, Wuhan 430079, China; The Innovation Research Center for Aquatic Mammals, Institute of Hydrobiology, Chinese Academy of Sciences, Wuhan 430072, China; Biodesign Center for Mechanisms of Evolution, Arizona State University, Tempe, AZ 85287, USA; Department of Biological Sciences & Environmental Change Initiative, University of Notre Dame, Notre Dame, IN 46556, USA; Biological Sciences, University of South Carolina, Columbia, SC 29208, USA; Biodesign Center for Mechanisms of Evolution, Arizona State University, Tempe, AZ 85287, USA; Key Laboratory of Pesticide & Chemical Biology of Ministry of Education, School of Life Sciences, Central China Normal University, Wuhan 430079, China

**Keywords:** *Daphnia obtusa*, mutation rate, gene conversion, loss-of-heterozygosity, natural selection

## Abstract

Spontaneous mutations are the primary source of genetic variation and play a central role in shaping evolutionary processes. To investigate mutational dynamics in *Daphnia obtusa*, we generated a chromosome-level genome assembly spanning 129.4 Mb across 12 chromosomes, encompassing 15,321 predicted protein-coding genes. Leveraging whole-genome sequencing of eight mutation-accumulation (MA) lines propagated for an average of 482 generations (spanning over 20 years), we estimated a spontaneous single-nucleotide mutation (SNM) rate of 2.45 × 10^−9^ and an indel mutation rate of 3.34 × 10^−10^ per site per generation. The SNM spectrum was strongly biased toward C:G > T:A transitions. Despite the design of MA experiments to minimize selection, nonsynonymous mutations were strongly underrepresented, providing rare evidence that purifying selection can act detectably even during mutation accumulation. Comparative analyses with natural population data revealed that exonic mutations observed in the MA lines were significantly less likely to be present in standing variation than intronic or intergenic mutations, suggesting that purifying selection in natural populations acts to remove deleterious alleles. We also identified 48 loss-of-heterozygosity (LOH) events, comprising 8 heterozygous deletions and 40 gene-conversion events, yet found no evidence of GC-biased gene conversion. Instead, while mutation predicts a substantially lower equilibrium GC content, the observed GC level is maintained at higher values, implicating natural selection as the primary force stabilizing base composition. Together, these results provide one of the most comprehensive assessments of the interplay among mutation, selection, and genome stability in an ecologically important species.

## Introduction

Because spontaneous mutations are the ultimate source of genetic variation, understanding the rate and spectrum of mutations is essential for addressing fundamental questions in molecular evolution, genetic disease, and genome-architecture dynamics (Lynch et al. 2016). Mutation rates influence both the adaptive potential of populations and the accumulation of deleterious load, making them among the most critical parameters in evolutionary biology. However, because de novo mutations are rare and often context-dependent, direct measurement requires long-term experimental designs that minimize the confounding effects of natural selection.

Mutation accumulation (MA) experiments, in which organisms are propagated through repeated single-progeny descent to reduce the efficacy of selection, offer a powerful means to estimate spontaneous mutation rates in vivo (Halligan and Keightley 2009; Katju and Bergthorsson 2019; Lynch et al. 2023). Nevertheless, most MA studies to date have focused on haploid microbes or obligately sexual species maintained under inbreeding regimes. These systems can exclude mutations such as recessive lethals, large-scale structural variants, and loss-of-heterozygosity (LOH) events. Moreover, the relatively short timescales and limited lineage diversity of many MA studies raise concerns about their ability to capture the full mutational spectrum, particularly for rare and complex mutation types (Baer et al. 2007; Narasimhan et al. 2017; Sasani et al. 2019).

The freshwater microcrustacean genus *Daphnia* is an emerging model for evolutionary and ecological genomics, with a short generation time, facultative sexual reproduction, and central role in aquatic food webs (Colbourne et al. 2011; Miner et al. 2012; Ebert 2022). Previous research on *Daphnia pulex* and *Daphnia magna* has revealed moderate base-substitution mutation rates in both species (Keith et al. 2016; Flynn et al. 2017; Ho et al. 2020). For *D. pulex*, the estimated mutation rates range from 2.30 × 10⁻^9^ to 4.53 × 10⁻^9^ substitutions per site per generation (Keith et al. 2016; Flynn et al. 2017), while the estimate for *D. magna* is somewhat higher, ∼8.9 × 10⁻^9^ per site per generation (Ho et al. 2020). However, the extent to which mutation rates and spectra vary across *Daphnia* species remains poorly understood.

To help fill this gap, we generated a chromosome-level genome assembly of *Daphnia obtusa,* a close relative of *D. pulex* that shares a similar life history and ecological niche, often co-occurring in small, temporary, fishless ponds. It provides an excellent system for comparative analysis due to its well-defined genetic and morphological distinctions. *D. obtusa* is widespread in North American ponds and lakes and reproduces exclusively via cyclical parthenogenesis (Hebert 1987). Morphologically, *D. obtusa* differs slightly from *D. pulex*: females possess large antennular mounds and weak carapace spinulation, while males are characterized by a short postabdominal process and the absence of a postabdominal bay. The presence of hairs on the inner lip of the carapace in females further distinguishes *D. obtusa* from *D. pulex* (Hebert and Crease 1983; Hebert and Finston 1996). Genetically, *D. obtusa* is moderately divergent from the *D. pulex-D. pulicaria* species complex, with a silent-site divergence of ∼0.134 (Ye et al. 2025), making it a highly useful outgroup in population-genomic studies of the latter (Maruki et al. 2022; Ye et al. 2022, 2023). This combination of a shared ecological context with a clear genetic and morphological divergence makes *D. obtusa* an ideal candidate for exploring the evolutionary dynamics of mutation in a comparative framework.

As a foundation for studying mutation and genome evolution in this lineage, we assembled the first chromosome-level reference genome for *D. obtusa*. We then performed deep whole-genome sequencing on eight independently maintained MA lines, each propagated asexually from a single clone for about 500 generations, representing one of the longest-running MA experiments in a multicellular eukaryote. This extensive temporal scale enables the accurate estimation of rare spontaneous mutations, including base substitutions, insertions/deletions (indels), and LOH events, as well as the identification of underlying mechanisms such as gene conversion and heterozygous deletion. Finally, by integrating our MA-derived mutational data with genome-wide polymorphism patterns from natural *D. obtusa* populations, we assess the extent to which natural selection shapes mutational outcomes. Together, our results provide a comprehensive view of the mutation and selective landscape in the genome of *D. obtusa*.

## Results

### Chromosome-level genome assembly and functional annotation

**Figure 1:**
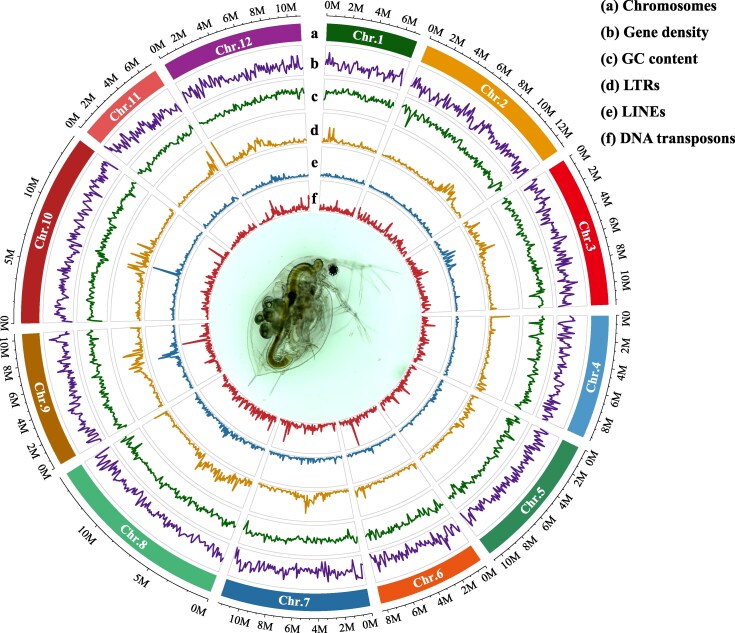
Genomic characteristics of *D. obtusa*. From the outermost to the innermost circle: a) the 12 chromosomes of the *D. obtusa* genome shown at the megabase (Mb) scale; b) gene density; c) GC content; d) long terminal repeats (LTR); e) long interspersed nuclear elements (LINE); and f) DNA transposons, each calculated using a 100 kb sliding window.

To obtain a chromosome-level genome assembly for *D. obtusa*, we used a clone collected from Illinois, USA, and clonally propagated in the laboratory. A total of 12.8 Gb (∼95×) PacBio reads were generated and assembled using Canu v1.4 (Koren et al. 2017). Multiple steps were applied to filter out non-*Daphnia* sequences (potential contamination from food and/or epibionts), e.g. removing contigs with low coverage and abnormal GC content (see Methods). The remaining contigs were scaffolded using 108× reads from high-throughput chromosome conformation capture (Hi-C) technology. The primary draft genome assembly presented here, FS6_V2, consists of 129.4 Mb of DNA sequences located on 12 chromosomes (Fig. 1). The contig N50 of the assembly is 984 kb and the scaffold N50 is 11.5 Mb, indicating high contiguity. The BUSCO completeness score (Simão et al. 2015) is 97.0%, with a duplication rate of 0.6%. The average GC content is 0.409, and approximately 24% of the genome consists of repetitive sequences, such as LTR retrotransposons, LINEs, as well as DNA transposons and tandem repeats (Table S1). Among them, LTRs, LINEs, SINEs, and DNA transposons account for 2.91%, 0.54%, 0.38%, and 0.97% of the genome, respectively.

To assist gene prediction, we generated a comprehensive transcriptomic dataset, including 73.1 million Illumina RNA-seq reads and 2.3 million full-length Isoseq reads derived from six environmental stress conditions (high temperature, Atrazine, NaCl, nickel, low pH, and UV light). Using a combined de novo and evidence-based annotation strategy (see Methods), we predict a total of 15,312 protein-coding genes (PCGs) and 5,216 long non-coding RNA (lncRNA) genes (Table S2). Of all PCGs, 94.4% are supported by transcriptomic evidence, whereas only 842 are predicted solely through ab initio methods. Among these 842 genes, 384 are absent in *D. pulex*. Functional annotation was successfully assigned to 11,188 PCGs using Blast2GO, representing 73% of the total.

The genome size of *D. obtusa* is very similar to that of *D. pulex* (129.4 Mb vs. 133.2 Mb). Similarly, the PCGs counts are nearly identical between the two species, with 15,321 in *D. obtusa* and 15,282 in *D. pulex* (Table S2), although only 11,230 orthologous genes are shared between them. Notably, the number of predicted lncRNA genes is slightly higher in *D. obtusa* (5,216 vs. 4,132). In terms of base composition, the GC content is nearly the same (40.9% vs. 40.6%). However, the content of repetitive elements is somewhat higher in *D. obtusa* (30.7 Mb vs. 25.7 Mb). Overall, these two species exhibit a high degree of similarity in genome content. The average silent-site divergence between them is 0.134, whereas the average divergence for nonsynonymous sites is 0.057 (Ye et al. 2025). The synteny map reveals only modest levels of genome rearrangement between the two species (Figure S1).

### Spontaneous mutation rates and mutational spectra

To estimate mutation rates in *D. obtusa*, we performed deep whole-genome sequencing on eight mutation-accumulation (MA) lines. These lines all descend from a single clone collected in November 2001 from a pond in Trelease Woods, Urbana, Illinois, USA. At the start of the MA experiment, 50 lines were initiated by isolating offspring from a single individual, and these lines were maintained under MA conditions for over 20 years. The eight lines that survived for deep whole-genome sequencing had been propagated asexually for an average of 482 generations (Table 1). Each sample yielded an average of 67 million uniquely mapped reads, with a mean coverage breadth of 84.7% and depth of 79× (Table S3). To ensure high-confidence variant calling, we restricted our analysis to genomic sites with read depths between 20 and 300, resulting in an average of 87 million callable sites per sample (Table S4).

**Table 1:** Mutation events detected in the MA lines.

Sample ID	Generation	Coverage	SNM	Indel	µ(SNM) × 10^−9^	µ(Indel) × 10^−10^
13-507	507	79	112	6	1.26	0.68
14-465	465	135	98	3	1.17	0.36
20-479	479	57	246	33	3.08	4.13
26-460	460	84	194	27	2.40	3.33
27-508	508	86	164	21	1.83	2.34
28-503	503	49	182	18	2.23	2.21
33-427	427	85	208	24	2.76	3.19
48-504	504	56	259	48	3.11	5.77
Average	482	79	183	23	2.23	2.75

The number before the hyphen in the sample ID represents the clone, while the number after The hyphen represents the generation number. SNM, single nucleotide mutation; Indel, small (<5 bp) insertions and deletions.

Variants were called using GATK (McKenna et al. 2010), and only high-confidence polymorphic sites were retained for downstream analysis. Across the *D. obtusa* genome, 99.43% of sites were homozygous in the MA lines, with only 0.57% being heterozygous. Because all lines were maintained clonally, ancestral heterozygous sites are expected to be preserved unless affected by events such as gene conversion, deletion, or single-nucleotide substitution. We first applied a series of stringent filters to identify and curate a reliable set of heterozygous sites, which represent potential variable positions. On average, each line initially contained approximately 0.50 million heterozygous sites, for which we used a binomial test to assess whether allelic ratios conformed to the expected 1:1 distribution. Additional filters included thresholds for strand bias, mapping quality, read position quality, and requiring at least five supporting reads for the alternative allele (see methods). After filtering, an average of ∼0.41 million high-confidence heterozygous sites and ∼86.8 million homozygous sites per line were retained (Table S4). After establishing this high-confidence set of background genotypes, we proceeded to identify de novo mutations. Based on the assumption that parallel mutations are extremely rare, we defined a de novo mutation as a site that was unique to a single MA line and represented a change from the ancestral homozygous state to a heterozygous state.

**Figure 2:**
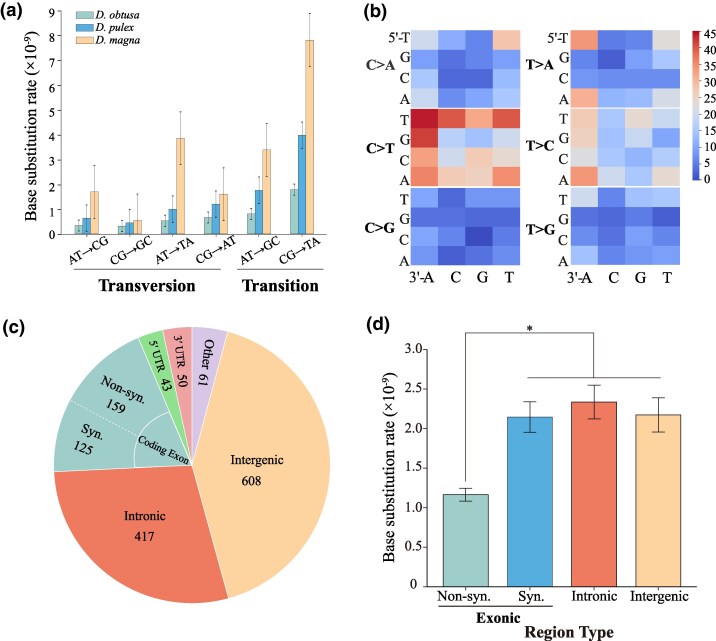
Patterns and distribution of spontaneous mutations in *Daphnia obtusa.* a) Conditional base mutation rates are compared among *D. obtusa*, *D. pulex* (Keith et al. 2016), and *D. magna* (Ho et al. 2020; averaged across genotypes). b) The context-dependent mutational spectrum shows the distribution of single-nucleotide mutations by trinucleotide context. c) Pie chart showing the distribution of mutations across different genomic compartments, including intergenic, exonic, and intronic regions. d) Mutation rates in intergenic, intronic, and exonic regions are compared. Statistical significance was tested using the *χ*^2^ test; *P < 0.05.

We identified a total of 1,463 single-nucleotide mutations (SNMs) and 180 insertions/deletions (≤5 bp) across all MA lines (File). Each line harbored between 98 and 259 SNMs and between 3 and 48 indels (Table 1). The base substitution mutation rate for each line was calculated as: *µ* = *x*/(*g* × 2*n*), where *x* represents the number of mutations, *g* represents the number of MA generations, and *n* is the number of callable sites (2*n* accounts for a diploid genome, and the fact that we are identifying heterozygous mutations). The overall SNM rate is estimated to be 2.23 × 10^−9^ mutations per base per generation (95% CI: 1.70 to 2.76 × 10^−9^), while the indel rate is 2.75 × 10^−10^ per base per generation (95% CI: 1.52 to 3.99 × 10^−10^). The SNM rate is close to the previous estimates in *D. pulex*, which range from 2.30 × 10^−9^ (Flynn et al. 2017) to 4.53 × 10^−9^ (for sexual clones, as reported in Table 1 of Keith et al. 2016). Among the base substitutions, the most frequent type is C:G > T:A, occurring at a frequency 5.6× higher than the least common type, C:G > G:C (Fig. 2a; Table 2). The transition-to-transversion (Ts/Tv) ratio is 1.31 (Table S5), notably higher than the null expectation of 0.5. This mutational spectrum contrasts with that reported in *D. pulex* by Flynn et al. (2017), who found C:G > A:T to be the most frequent substitution and a Ts/Tv ratio of 0.81. However, our results closely align with those for *D. pulex* reported by Keith et al. (2016), who also observed C:G > T:A as the dominant mutation type and a Ts/Tv ratio of 1.58 (Fig. 2a), and data from *D. magna* (Ho et al. 2020) reveal a comparable mutational pattern, with C:G > T:A as the most common substitution and a Ts/Tv ratio of 1.54 (Fig. 2a). We also examined multinucleotide mutations (MNMs), defined as mutations occurring within 50 bp of each other, and identified 93 such events, accounting for approximately 6.4% of all base substitutions. To estimate the expected GC content of the genome at mutation equilibrium under neutrality, we used the rate of G/C → A/T substitutions (*v*) and the rate of A/T → G/C substitutions (*u*). The equilibrium GC-content reflects the long-term balance between GC losses and gains and is calculated as *u/(u*  *+*  *v)*. This model assumes that the nucleotide composition has reached a steady state in which the rate of GC loss equals the rate of GC gain. Based on this calculation, the equilibrium GC content under mutation pressure from base substitutions alone is estimated to be 0.320, which is much lower than the observed GC content of 0.409.

**Table 2:** Conditional mutation rates of all six possible substitution types.

Types	Substitution	Frequency	Mutation rate (/site/generation) × 10^−10^
Transition	C:G →T:A	497	18.53
	A:T →G:C	332	8.59
Transversion	A:T →T:A	215	5.54
	C:G→A:T	186	6.93
	A:T →C:G	144	3.71
	C:G→G:C	89	3.29

Given the very large mutational harvest, to investigate context-dependent patterns of spontaneous mutations, we analyzed the 96-trinucleotide mutation spectrum, which considers the six types of base substitutions across all 16 possible combinations of flanking 5′ and 3′ nucleotides. We found that substitution rates in *D. obtusa* are significantly influenced by the identity of the central base and its neighboring nucleotides. Specifically, mutations were 1.12 times more frequent when the central base was a C (or G on the complementary strand) than when it was an A or T, highlighting a nucleotide-specific bias. For the most common mutation type, C > T transitions, there is a strong influence of local sequence context. These mutations occurred most frequently when the focal cytosine is preceded by a T at the 5′ position or followed by an A at the 3′ position (Fig. 2b), suggesting that certain flanking bases enhance mutability at cytosine sites. 5′T–C–A3′ is one of the classic target motifs of APOBEC deaminases, where the flanking 5′T and 3′A context makes the central cytosine more susceptible to deamination into uracil. If this uracil is not repaired before DNA replication, the replication machinery interprets it as thymine (T) and incorporates an adenine (A) opposite it. As a result, the original C:G base pair becomes a T:A base pair in the daughter DNA strand. Over time, this leads to a stable C to T transition mutation in the genome. Interestingly, CpG sites generally show reduced mutation rates, with the exception of C > T transitions, which show elevated mutation frequencies. These results underscore the significant role of local sequence context in shaping the mutational landscape of the genome.

We next assessed mutational bias across different genomic compartments, including intergenic, exonic, and intronic regions. Among the 1,463 SNMs identified, 608 occurred in intergenic regions, 417 in introns, and 284 in coding exons (Fig. 2c). To evaluate whether mutation rates differ among these functional categories, we normalized the mutation counts by their respective genomic proportions. Our analysis revealed that introns, intergenic regions, and synonymous sites within exons exhibit a comparable density of accumulated mutations. In contrast, we observed a significant deficit of mutations at nonsynonymous sites (Fig. 2d; *χ*^2^ > 3, *P* < 0.05 for all comparisons involving nonsynonymous sites). This pattern is not consistent with a uniform accumulation of mutations across all site types. This finding strongly suggests the action of purifying selection, even within the context of our MA experiment. Although the experimental design minimizes the efficacy of selection, deleterious mutations are likely to have been purged from the surviving lines and may have contributed to the extinction of a substantial proportion of lines. Because nonsynonymous mutations are more likely than synonymous mutations to be deleterious, this process explains their underrepresentation in the final dataset. Therefore, the reduced frequency of observed nonsynonymous mutations is best interpreted as evidence of strong evolutionary constraint at these sites, rather than a lower intrinsic mutation rate.

As 21% of the genomic sites correspond to nonsynonymous positions, and the observed mutation rate at these sites is on average only 53% of that observed in other categories (intronic, intergenic, and synonymous sites), the nonsynonymous mutation rate was underestimated by 47%. To correct for this bias, we adjusted the overall mutation rate as follows: Corrected rate = (0.21 × (1 + 0.47) × *µ*) + (0.79 × *µ*), where *µ* represents the raw mutation rate, 0.21 is the proportion of nonsynonymous sites. Given a spontaneous SNM rate of 2.23 × 10^−9^, the corrected estimates are approximately: 2.45 × 10^−9^. Indels in coding regions are expected to be even more strongly depleted due to their higher deleterious effects. The observed indel rate in exonic regions (1.08 × 10^−10^) is about 29% of the average rate in intronic and intergenic regions (4.14 × 10^−10^ and 3.26 × 10^−10^, respectively) (Figure S2), indicating a 71% underestimation. Assuming the true mutation rate is uniform across regions and that exonic sites constitute about 30% of the genome, the corrected indel mutation rate is estimated at 3.34 × 10^−10^ per site per generation.

To further investigate the potential functional consequences of SNMs, we annotated their predicted impacts using SnpEff (Cingolani et al. 2012). Of all identified SNMs, 2.0% were classified as high-impact variants, typically protein-truncating mutations such as stop-gained. Approximately 13.9% were predicted to be moderate impact, generally representing missense mutations. Another 8.6% were annotated as low impact, corresponding to synonymous substitutions. The remaining majority (∼75%) were categorized as potential modifiers, referring to variants located in noncoding regions such as introns, untranslated regions (UTRs), or intergenic sequences (Table S6). To evaluate whether these proportions deviate from neutral expectations, we performed 1,000 simulations of the same number of SNMs, maintaining the observed transition-to-transversion ratio, and annotated them using SnpEff to generate a neutral distribution of functional categories. The proportion of high-impact variants among observed SNMs (2%) was slightly lower than the neutral expectation (2.8% ± 0.10%) (Table S6), consistent with the loss of strongly deleterious mutations during the MA process. Notably, moderate-impact variants, which generally correspond to missense mutations, were observed at 13.9%, substantially lower than the neutral expectation of 23.7% ± 0.26% (Table S6), indicating that these variants are under strong purifying selection. In contrast, low-impact variants were observed at 8.6% versus 9.2% ± 0.19% under neutrality, suggesting that selection is relatively weak on these mutations. Finally, to assess whether SNMs are evenly distributed across chromosomes, we examined their chromosomal distribution. While chromosome 11 showed slightly elevated mutation rates, a *χ*^2^ test indicated no significant deviation from a uniform distribution (Figure S3; *χ*^2^ = 2.02, df = 11, *P* = 0.989).

**Figure 3:**
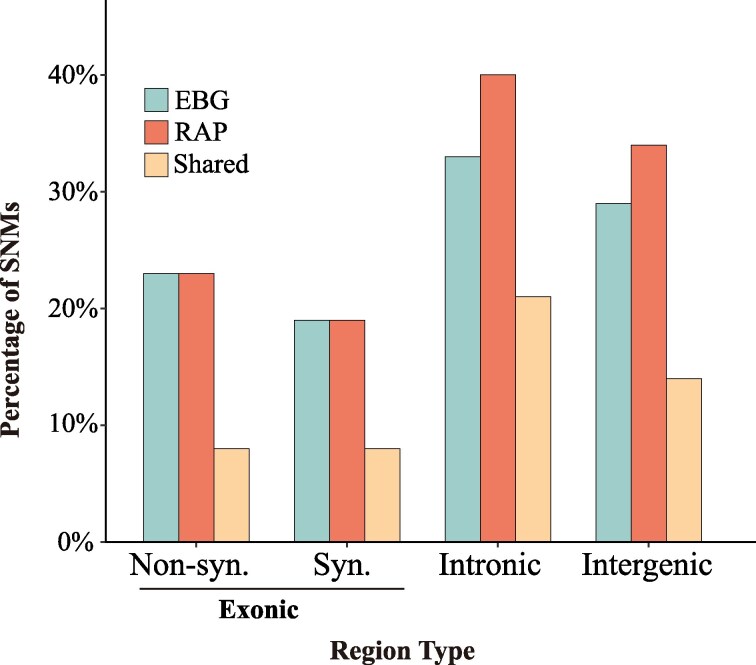
Percentage of SNMs from the MA lines detected as SNPs in two natural populations (EBG and RAP), shown across genomic categories: exonic, intronic, and intergenic regions.

To investigate how the mutational spectra observed in MA lines compare to segregating variants in natural populations of *D. obtusa*, where selection is acting, we sequenced two natural populations (EBG and RAP), each comprising over 90 unique genotypes (Table S7). Using a minor-allele frequency (MAF) threshold of >0.02 to define the presence of alternative alleles, we found that 337 (23%) and 393 (27%) of the SNMs from the MA lines were also present as SNPs in two reference populations (EBG and RAP) (Fig. 3). This indicates that a comparable fraction of mutations arising in the MA experiment are also represented as standing variation in natural populations.

Genomic compartment analysis indicated that the likelihood of MA-derived mutations being observed as segregating SNPs in natural populations differs across functional categories. On average, 32% and 36% of the SNMs from the MA experiment were also detected as SNPs in intronic and intergenic regions, respectively, whereas only 22% and 20% of SNMs were recovered as SNPs in nonsynonymous and synonymous coding sites (Fig. 3; Table S8). This pattern indicates a relative depletion of coding-region mutations among segregating variants, consistent with stronger selective constraints in functional regions.

We further estimated the effective population size (*N_e_*) of the EBG and RAP populations using the formula *N_e_*  *=*  *π_s_/4μ*, where *μ* is the mutation rate (2.45 × 10^−9^ per site per generation) and *π_s_* is the nucleotide diversity at synonymous sites. Based on *π_s_* values of 0.017 for RAP and 0.012 for EBG, we estimated *N_e_* values of approximately 1,735,000 and 1,224,000, respectively. These estimates are substantially higher than those reported for *D. pulex* (*N_e_* ≈ 640,000; Lynch et al. 2017) and *D. magna* (*N_e_* ≈ 418,000; Ho et al. 2020). The mutation rate in *D. obtusa* (2.45 × 10^−9^) is comparable to the lower estimate in *D. pulex* (2.30 × 10^−9^; Flynn et al. 2017), lower than the higher estimate for sexual clones in *D. pulex* (4.53 × 10^−9^; Keith et al. 2016), and markedly lower than that of *D. magna* (8.9 × 10^−9^; Ho et al. 2020). These relationships are qualitatively consistent with predictions of the drift-barrier hypothesis, which posits that species with larger *N_e_* tend to evolve lower mutation rates due to more efficient selection against mutator alleles, although such differences can simply arise stochastically as mutation rates drift around the drift barrier (Lynch 2011; Lynch et al. 2016).

### Loss of heterozygosity

Loss of heterozygosity (LOH), defined as the transition from a heterozygous to a homozygous state within a specific genomic region, plays a critical role in evolutionary genetics. LOH can arise via various mechanisms, including gene-conversion-like processes (during mitotic cell divisions, in this case) and heterozygous deletion. To identify de novo LOH events, we employed the Runs of Homozygosity (ROH) module implemented in BCFtools (Narasimhan et al. 2016), which applies a hidden Markov model (HMM) to detect homozygous-by-descent tracts based on genotype likelihoods from VCF files. Candidate LOH regions were defined as contiguous tracts with high HMM confidence scores, which consistently contained heterozygous sites in nontarget MA lines. Using this criterion, we identified a total of 48 LOH events, with the number of events per MA line ranging from 0 to 22 (Table 3; File). The minimal span of these LOH regions ranged from 1.1 kb to 2.45 Mb, while the maximum size range extended from 1.6 kb to 2.49 Mb (File). Here, the minimum size refers to the distance between the first and last homozygous SNPs within a given LOH event, whereas the maximum size refers to the distance between the first and last homozygous SNPs while also encompassing the flanking heterozygous sites. We estimated a genome-wide LOH rate of 2.93 × 10^−5^ per heterozygous site per generation. This estimate is broadly consistent with a previous report for *D. pulex* (4.82 × 10^−5^; Flynn et al. 2017), though somewhat lower than that reported by Omilian et al. (2006), who estimated a rate of 1.8 × 10^−4^ per heterozygous site per generation. However, direct comparisons across studies should be interpreted with caution due to differences in experimental design and methodological approaches.

**Table 3:** Summary of LOH (loss-of-heterozygosity) events across MA lines.

Sample ID	Events	Mean length (kb)	Mean sites	LOH rates
13-507	1	231 (NA)	264 (NA)	1.66 × 10^−6^
14-465	0	NA	NA	NA
20-479	5	90 (57)	248 (143)	8.36 × 10^−6^
26-460	6	404 (205)	578 (226)	2.40 × 10^−5^
27-508	1	412 (0)	1,595 (0)	1.02 × 10^−5^
28-503	8	397 (302)	1,001 (659)	5.60 × 10^−5^
33-427	5	38 (38)	164 (140)	6.05 × 10^−6^
48-504	22	338 (85)	869 (243)	1.36 × 10^−4^
Total	48	14,324	34,538	2.93 × 10^−5a^

The LOH rate for each mutation accumulation (MA) line was calculated using the formula: *µ* = *x*/(*g* × *n*), where *x* is the number of LOH sites observed, *g* is the number of MA generations, and *n* is the number of heterozygous sites in the ancestral genotype. Mean LOH tract lengths (the distance between the first and last homozygous SNPs within a given LOH event) and the number of sites per event are shown with their corresponding standard errors in parentheses.

^a^The total conversion rate was calculated as the average rate across all eight MA lines.

To investigate the mechanisms underlying the LOH, specifically whether they result from heterozygous deletions or gene conversion, we analyzed sequencing depth across LOH regions. Regions exhibiting approximately 50% of the sequencing coverage relative to flanking regions and other MA lines were classified as heterozygous deletions. In contrast, LOH regions with coverage similar to their surrounding genomic context were considered consistent with gene conversion-like processes, such as ameiotic recombination. Among the 48 identified LOH events, 8 were attributed to deletions, while 40 were attributed to gene conversion (File). The average length of conversion tracts was 320 kb. The estimated genome-wide rate of conversion-induced LOH was 2.62 × 10^−5^ per heterozygous site per generation (Table S9). This rate is reasonably comparable to that observed in *D. pulex* lines (6.47 × 10^−6^; average across obligately asexual and cyclically parthenogenetic *D. pulex* in Keith et al. 2016). However, due to the high variability in the *D. magna* data (Ho et al. 2020), direct comparisons are not feasible.

To examine whether gene conversion exhibits transmission bias, we tracked the outcome of each converted heterozygous site as either an A/T or a G/C allele. Converted sites showed a slight excess of GC alleles (50.2%), but this did not deviate significantly from an equal 1:1 ratio (binomial test; *P* = 0.506), indicating that gene conversion is not biased toward GC.

## Discussion

This study represents a substantial advance in our understanding of the mutational processes and evolutionary dynamics of *Daphnia obtusa*, and, owing to the extensive mutational data generated, it also provides insights of broader relevance to invertebrates in general. By combining three uniquely valuable genomic resources—a high-quality chromosome-level genome assembly, over two decades of MA data, and population-genomic data from natural populations—we provide an integrative view of how spontaneous mutations arise and are shaped by natural selection. These datasets together form a valuable platform for exploring evolutionary processes in a key aquatic model organism.

Our estimation of the spontaneous mutation rate (*μ*) in *D. obtusa* falls squarely within the range reported for other eukaryotes, particularly invertebrates (Lynch et al. 2023). We calculated *μ* to be approximately 2.45 × 10^−9^ per site per generation, in the range of prior estimates in *D. pulex* (2.30 × 10^−9^ to 4.53 × 10^−9^; Keith et al. 2016; Flynn et al. 2017), *Caenorhabditis elegans* (2.7 × 10^−9^; Denver et al. 2009), and *Drosophila melanogaster* (2.8–5.49 × 10^−9^; Schrider et al. 2013; Keightley et al. 2014). The observed mutational spectrum, with a strong bias toward C:G > T:A transitions, reflects a conserved pattern found in diverse animals. For instance, a similar transitional bias is a dominant feature of the spontaneous mutation landscape in both humans (Jónsson et al. 2017) and *D. melanogaster* (Schrider et al. 2013). Although this bias is often attributed to methylation and subsequent cytosine deamination, *Daphnia* exhibit minimal DNA methylation largely restricted to gene bodies (Asselman et al. 2015), suggesting that alternative mutagenic processes may be responsible. Moreover, indels occur at approximately one-eighth the frequency of SNPs, are mostly short (<5 bp), and are enriched in repetitive or low-complexity regions—characteristics indicative of replication slippage (Tautz and Schlötterer 1994). Additionally, we observed a Ts:Tv ratio of approximately 1.31, consistent with the universal trend of transitions being more frequent than transversions due to the molecular mechanisms of base misincorporation and repair (Kunkel and Erie 2005; Lynch et al. 2023).

We next assessed mutational patterns across different genomic compartments and found that, after normalizing by genomic content, mutation rates were broadly similar among intergenic regions, introns, and synonymous coding sites. In contrast, nonsynonymous sites exhibited a significant deficit of mutations. The lower proportion of nonsynonymous single-nucleotide mutations observed in *D. obtusa* likely reflects the combined effects of residual purifying selection and developmental filtering inherent to MA experiments. Although MA experiments are designed to minimize selection through repeated bottlenecking and reduced effective population size, purifying selection is rarely eliminated completely. Mutations with large deleterious effects can still be efficiently purged even under MA conditions (Katju and Bergthorsson 2019). This interpretation is supported by the high extinction rate observed in our experiment, where 42 out of 50 *D. obtusa* MA lines failed to survive, likely because lines carrying strongly deleterious mutations—such as nonsynonymous ones—were preferentially lost. However, it should be noted that over the course of 20 years, and movement across labs, some lines were likely lost via nonselective events. Moreover, early developmental selection may remove deleterious germline mutations before transmission, as shown in multicellular eukaryotes where many such mutations are eliminated prior to sampling (Schrider et al. 2013; Keightley et al. 2014). Moreover, subtle selection acting during clonal propagation can bias the recovered mutation spectrum toward less deleterious variants (Mahilkar et al. 2022). Together, these findings suggest that the reduced fraction of nonsynonymous SNMs in *D. obtusa* primarily reflects residual purifying selection, prezygotic loss of deleterious mutations, and selective constraints maintaining genomic base composition. Therefore, the reduced frequency of observed nonsynonymous mutations is best interpreted as evidence of strong evolutionary constraint on these protein-coding regions, rather than a lower intrinsic mutation rate. This interpretation is further supported by the functional annotation of the observed mutations.

Among all single nucleotide mutations, only ∼2% were classified as high-impact (e.g. stop-gained), 13.9% as moderate-impact (missense), and the majority (∼84%) were annotated as low-impact or noncoding variants. The proportion of high-impact mutations (2%) was slightly lower than the neutral expectation of 2.8% ± 0.10%, which likely reflects both their intrinsic rarity in the genome and the strong purifying selection acting even under MA conditions, as suggested by the high extinction rate of MA lines. Moderate-impact mutations occurred at 13.9%, substantially below the neutral expectation of 23.7% ± 0.26%, indicating that these nonsynonymous variants are efficiently purged due to their functional consequences. In contrast, low-impact mutations, primarily synonymous substitutions, were observed at rates similar to neutrality (8.6% versus 9.2% ± 0.19%), suggesting weak or negligible selection. The observed scarcity of functionally significant mutations, together with the pronounced deficit of nonsynonymous substitutions, reinforces the conclusion that strong purifying selection continues to act even under MA conditions. In contrast, the comparable mutation densities across noncoding and synonymous sites indicate that the underlying mutational process is relatively uniform. This contrast makes the depletion of nonsynonymous mutations the clearest signal of selective constraint during the MA phase, highlighting how residual selection shapes the observed mutation spectrum.

Our comparison of MA-derived mutations with standing variation in natural populations provides insights into the selective forces shaping genomic diversity. Approximately one third of the SNMs identified in the MA experiment were also present as segregating SNPs in the RAP and EBG populations. Their distribution, however, was not uniform across genomic compartments. Exonic mutations, particularly nonsynonymous ones, were less frequently retained than intronic or intergenic mutations, a pattern consistent with purifying selection acting to remove functionally deleterious variants from natural populations. However, interpreting these patterns requires caution. The absence of a given SNM in a natural population does not necessarily indicate selection; it may instead reflect demographic factors, lineage sorting, or the mutation's absence in ancestral lineages. Interestingly, synonymous SNPs were not retained at a higher rate than nonsynonymous ones. While this observation might be suggestive of weak selective pressures such as codon usage bias, mRNA stability, or translational efficiency (Chamary and Hurst 2005; Plotkin and Kudla 2011), it should be interpreted with caution. Given that most genomic sites in natural populations are monomorphic and our sample size is limited, the probability of detecting the same mutation in both the MA experiment and natural populations is inherently low, regardless of selection. Thus, while our results are broadly consistent with the action of purifying selection, alternative demographic and stochastic explanations cannot be excluded.

Finally, we examined the mutation spectrum's implications for base composition evolution. The mutational spectrum in *D. obtusa* is strongly AT-biased, primarily due to frequent C:G > T:A transitions, which would be expected to reduce genomic GC content over time in the absence of counteracting forces. Although GC-biased gene conversion (gBGC) has been proposed as one such mechanism, our analysis of the observed conversion events revealed no significant GC bias, indicating that gene conversion in *D. obtusa* is effectively unbiased. However, this conclusion should be interpreted with caution. The effect of gBGC can be subtle, and our dataset of only 40 conversion events may lack the statistical power to detect weak biases. Moreover, studies in yeast have shown that GC-biased conversion is largely restricted to crossover-associated events (Lesecque et al. 2013). Because our data were generated using short-read sequencing, we cannot phase sufficiently long genomic segments to identify reciprocal exchanges in the flanking regions and therefore cannot determine whether the observed conversion events are crossover- or noncrossover-associated. It is possible that most of these events are noncrossover in nature, which would be consistent with the absence of a detectable GC bias. Thus, the apparent lack of GC bias in *D. obtusa* may reflect both limited sample size and the predominance of non-crossover events, rather than a true absence of bias, underscoring the need for caution when extrapolating these results to genome-wide base composition evolution.

Under the assumption of unbiased gene conversion, the equilibrium GC content is expected to be determined solely by mutation rates, predicting a value of 0.320, substantially lower than the observed genome-wide GC content of 0.409. This discrepancy suggests that selection, rather than mutation or conversion alone, plays a key role in maintaining elevated GC levels. In particular, selection may favor G/C nucleotides in functionally important regions where they contribute to amino-acid usage, structural stability, regulatory efficiency, and translational optimization. Thus, while mutation and conversion shape the raw input of genomic variation, natural selection likely acts as a stabilizing force preserving GC content in *D. obtusa*, especially in regions under functional constraint. Accordingly, highly expressed genes, which are typically under stronger selective constraints, appear to deviate more from the neutral GC expectation (Figure S4). We observed a strong positive correlation (*r* = 0.73) between gene expression level and ΔGC (the deviation of GC content from the neutral expectation), supporting the view that selection acts to preserve higher GC content in transcriptionally active regions. Moreover, genes harboring mutations did not exhibit higher expression levels than non-mutated genes (*P* = 0.083, Mann–Whitney *U* test; Figure S5), consistent with the notion that the observed GC bias is not simply a byproduct of transcription-associated processes but rather reflects genuine selective maintenance of GC-rich regions in *D. obtusa*.

## Materials and methods

### Genome assembly and annotation of *D. obtusa*

We first assembled the PacBio reads using Canu v1.4 (Koren et al. 2017) with default parameters. Then, multiple filters were applied to the primary assembly: (i) removed contigs with coverage lower than 10 × as they may represent assembly errors; (ii) removed contigs with abnormal GC content (<30% or >50%) due to eliminate potential bacterial or algal contamination; (iii) performed BLAST searches of each contig against the NCBI non-redundant (NR) database and discarded contigs with nonmetazoan origins; (iv) contigs with low coverage from Hi-C reads (5×) were removed from the assembly; (v) false inclusion of two allelic copies from the heterozygous regions were purged using purge_dups (Guan et al. 2020); (vi) removed mitochondrial contigs. The filtered contigs were then scaffolded into chromosomes using Hi-C interaction data. Hi-C contact maps were generated using Juicer (Durand et al. 2016a) and manually curated with Juicebox v1.11.08 (Durand et al. 2016b).

To estimate genome size, we used a k-mer based approach by creating a histogram from raw Illumina reads with Jellyfish 2.3 (Marçais and Kingsford 2011) and running the GenomeScope web application (http://genomescope.org/genomescope2.0/, last accessed November 2022). RepeatModeler 2.0 (Smit and Hubley 2015) was used to identify *D. obtusa-specific* repeats. Repeat sequences in the genome assembly were detected and masked using RepeatMasker-4.1.2-p1 (Smit et al. 2004) with default settings. Completeness in terms of single-copy core orthologs of the final scaffolds was assessed with BUSCO 3.0.2 (Simão et al. 2015), using the Arthropoda set (odb10). (Creation date: 2020-09-10, number of genomes: 90, number of BUSCOs: 1013).

To annotate the genome assembly, we exposed individuals to six abiotic perturbations linked to anthropogenic disturbance. These exposures included: standard and high temperatures (18 °C and 26 °C, respectively), low pH (5.0), UV light, nickel (0.03 g/L), Atrazine (4 mg/L), and sodium chloride (5 g/L NaCl). All treatments were maintained for 24 h period without additional algae, except for the UV treatment. The UV exposure was conducted in 250 ml beakers containing 50 ml of medium, placed 10.5 cm below 30 W, 36-inch Reptisun 5.0 UVB fluorescent bulbs for 4 h. All treatments, except high temperature, were conducted at 18 °C, and total RNA was extracted immediately afterward using either the RNeasy Mini Kit or Direct-zol RNA Miniprep Kit (Zymo, Cat. No. R2052). RNA samples were sent to the Translational Genomics Research Institute (TGen) in Phoenix for Illumina sequencing, and to UC Irvine for IsoSeq long-read sequencing. Prior to annotation, repeat sequences were masked as described above. Genome annotation was then carried out using the Eukaryotic Genome Annotation Pipeline-External (https://github.com/ncbi/egapx), which combines ab initio and evidence-based gene prediction. Functional annotation of predicted genes was performed using Blast2GO (Götz et al. 2008), allowing assignment of protein domains and Gene Ontology terms. To identify orthologous genes between *D. pulex* and *D. obtusa*, we used orthologous group data from OrthoDB v10 (Kriventseva et al. 2019). Protein sequences for each species were downloaded from NCBI and subsequently clustered into orthologous groups.

### MA line propagating and sequencing

All the MA lines used in this study descend from a single clone of *D. obtusa* collected from a pond in Trelease Woods in Illinois, USA (Latitude: 40.1297; Longitude: −88.1437) in the Spring of 2001. Fifty mutation accumulation (MA) lines were started in November 2001 by taking 50 offspring from a single individual. These 50 offspring became generation 1 of the lines. All MA lines were established and maintained with daily non-quantitative feeding with either *Scenedesmus obliquus* (in Indiana, USA) or *Ankistrodesmus falcatus* (in South Carolina, USA), in beakers with ∼100 mL filtered (1 mm) lake water, and kept in controlled environment chambers at 20 °C on a 12:12 L:D photoperiod.

The standardized procedure for propagating the experimental lines was as follows: 8 to 10 days following the start of the previous generation, a single randomly chosen female offspring was transferred to a new beaker of lake water. Maturation takes place after ∼7 days at 20 °C, which ensured that the transferred individual was a daughter and not a granddaughter. If a line had not produced offspring by the time of transfer, the mother was transferred to a new beaker and the generation number for that line was not increased. In addition to the focal individual transferred, two of her sisters were transferred into separate beakers to serve as backups. Backups were used for a transfer when the focal individual either died before reproducing or produced only male offspring and/or diapausing eggs over her entire life. Throughout the course of the project, backups were used in ∼10% of the transfers. Use of backups neither showed a trend over time nor was clustered in certain lineages (McTaggart et al. 2007). Approximately every fifth generation, sisters of the focal individual were collected and frozen at −80° for molecular analyses. Several steps were taken to minimize the risk of exogenous contamination and cross-contamination among the lines. Beakers were kept covered to prevent splash contamination when they were not in use. Pipettes used to handle the animals were changed after each transfer to avoid any neonates that may have adhered to the pipette.

Eight lines with a generation time close to 500 were survived for sequencing. DNA was extracted from around 100 to 500 clonal individuals using the MasterPure Complete DNA & RNA Purification Kit (Cat. No. MC85200) following the manufacturer's protocol. To minimize bacterial contamination, for any DNA collected here for sequencing libraries, we starved the culture for 2 d in COMBO media. DNA quality and Concentration were assessed by NanoDrop one and dsDNA HS Qubit Assay (Molecular Probes by Life Technologies, No. Q32851). The integrity of DNA was determined by TapeStation 4200 system. The DNA passed the quality test was sent to The Translational Genomics Research Institute at Phoenix for library construction and sequencing.

### Variants calling and spontaneous mutation rates

To ensure high-quality base-calling, we first removed sequencing adapters and trimmed low-quality bases from the raw reads using Trimmomatic v0.33 (Bolger et al. 2014). The cleaned reads were subsequently mapped to the *Daphnia obtusa* reference genome (FS6_V2) using HISAT2 v2.1.0 (Kim et al. 2019). To retain only uniquely mapped reads, we applied SAMtools v1.8 (Li et al. 2009) with the -q 60 option to exclude those aligning to multiple genomic locations. Variant calling was performed using the HaplotypeCaller module in GATK v4.2.5.0 (Van der Auwera and O’Connor 2020). To ensure high-confidence genotype calls, we applied a stringent filtering pipeline: (i) duplicated reads were removed using Picard MarkDuplicates function (http://broadinstitute.github.io/picard); (ii) We excluded SNPs or indels located within 20 base pairs of another indel; (iii) tri-allelic sites and multi-allelic sites were excluded from the analysis (∼3% of the total polymorphic sites); (iv) Heterozygous sites were retained only if the minor allele frequency (MAF) was ≥ 0.2; (v) only sites with sequencing depth between 20× and 300× were included in downstream analyses; (vi) Strand bias was assessed using Fisher's Exact Test, and sites with bias scores greater than 60 were filtered out; (vii) A Mann–Whitney *U*-based *z*-approximation was used to test for differences in mapping quality between reads supporting reference and alternate alleles; sites with values less than −12.5 were removed; (viii) We also used the Mann–Whitney Rank Sum Test to evaluate read position bias, excluding sites with scores below −8.0; (ix) a binomial test was applied to assess deviation from the expected 0.5:0.5 allele balance, and a hard filter was applied to the ALT read counts in the AD field, removing variants with fewer than five supporting reads.

To estimate the base substitution mutation rate, we first inferred the ancestral genotype at each polymorphic site, defined as the allele shared by at least seven MA lines. A de novo base substitution mutation was identified when one MA line exhibited a novel variant allele while the other lines retained the ancestral genotype. Mutations were excluded if they occurred within 20 bp of an indel or if any of the unmutated lines contained reads supporting the same variant allele, indicating potential pre-existing polymorphisms. Given that over 99% of the *D. obtusa* genome is homozygous, we focused exclusively on Hom > Het mutations, where a homozygous ancestral site becomes heterozygous in the mutated MA line. The nuclear base substitution mutation rate of each MA line was calculated as follows: *µ* = *x*/(*g* × 2*n*), where *x* represents the number of base substitution mutations that passed the filters, *g* represents the number of MA generations, and *n* is the number of callable sites (2*n* represents diploid genome).

We used the rate of G/C → A/T substitutions (***v***) and the rate of A/T → G/C substitutions (***u***) to estimate the expected GC content of the genome at mutation–equilibrium. To derive *u* and *v*, we classified all observed base substitutions into two categories: those converting A or T into G or C (AT→GC, contributing to *u*), and those converting G or C into A or T (GC→AT, contributing to *v*). The equilibrium GC content reflects the long-term balance between GC losses and gains and is calculated using the following formula: GCeq = *u*/(*u* + *v*).

### Mutation spectrum in natural populations of *D. obtusa*

In the spring seasons of 2014 and 2016, we collected *Daphnia obtusa* isolates from seven randomly selected ponds across the United States (Table S7). To increase the likelihood that each isolate originated from recently hatched resting eggs, adults were sampled early in the growth season. After propagating each isolate clonally in the laboratory, a high mortality rate in populations JP, AQP, and RZP resulted in an insufficient number of viable clones for sequencing. For the remaining populations, DNA was extracted from 96 isolates per population. Library preparation was conducted using the Nextera kit, with each sample labeled using a unique oligonucleotide barcode. Sequencing was performed on the Illumina HiSeq 2500 platform, generating paired-end 150 bp reads. To enhance our ability to detect signatures of selection, we aimed to capture genetically diverse individuals by sampling at the time of hatching from sexually produced resting eggs. However, in populations TRH and PYR, most clones appeared to be descendants of the same maternal lineage, as determined by the relatedness function in MAPGD (v0.4.26; Ackerman et al. 2017). This reduced genetic diversity and, consequently, the power to detect selection. Therefore, only populations RAP and EBG, each comprising more than 90 unique genotypes, were retained for downstream analyses.

To prepare data for population-genomic analyses, we constructed nucleotide-read quartets (counts of A, C, G, and T at each site) from raw FASTQ files. After removing adapter sequences with Trimmomatic (v0.36) (Bolger et al. 2014), we aligned the reads to the *D. obtusa* reference genome using Novoalign (v3.02.11) with the “-r None” setting to exclude multi-mapping reads. The resulting SAM files were converted to BAM format using Samtools (v1.3.1) (Li et al. 2009). We then marked duplicates and performed local realignment around indels using GATK (v3.4-0) (McKenna et al. 2010; DePristo et al. 2011; Van der Auwera et al. 2013) and Picard tools. To eliminate redundancy, overlapping read pairs were clipped with BamUtil (v1.0.13), and the final mpileup files were created using Samtools. We generated a comprehensive nucleotide-read profile using the proview command from MAPGD (v0.4.26) (Ackerman et al. 2017).

Despite these preprocessing steps, sequencing artifacts can still affect data accuracy. For instance, some reads may originate from unassembled paralogous regions, leading to anomalous signals at certain sites. To reduce the impact of such mismappings, we excluded any site where a goodness-of-fit test (Ackerman et al. 2017) identified non-binomial read distributions in four or more individuals. We further filtered out clones with mean genome-wide coverage below 3× or clones with total goodness-of-fit scores less than −0.4, which may indicate contamination or other quality issues. To remove potentially unreliable genomic regions, we filtered out sites overlapping with repetitive elements identified using RepeatMasker (v4.0.5) and a custom repeat library (Jurka et al. 2005, last updated August 7, 2022). Additional population-wide coverage thresholds were set to avoid artifacts from under- or over-represented loci. After completing all filtering steps, we estimated allele and genotype frequencies for each population using the maximum-likelihood method implemented in the *allele* function of MAPGD (Ackerman et al. 2017). Finally, sites with error-rate estimates greater than 0.01 were excluded from downstream analysis.

To investigate the mode of natural selection acting on amino-acid sequences of protein-coding genes, we calculated genetic diversity at synonymous (π_S_) and nonsynonymous (π_N_) sites for each gene in both populations, along with the π_N_/π_S_ ratio. For a given site in a specific population, π was estimated as 2*p*(1−*p*), where *p* represents the minor allele frequency obtained from the *allele* function of MAPGD (Ackerman et al. 2017).

### Loss of heterozygosity

To identify loss of heterozygosity (LOH) events in MA lines, we systematically analyzed heterozygous sites across all genotypes. De novo LOH events were detected using the ROH module in BCFtools (Narasimhan et al. 2016), which employs a hidden Markov model (HMM) to identify homozygous-by-descent tracts based on genotype likelihoods from VCF files. Accurate estimation of runs of homozygosity (ROH) in BCFtools requires an external allele frequency (AF) file derived from population-level variant data. We generated this input using the maximum-likelihood method implemented in the allele function of MAPGD (Ackerman et al. 2017). Allele frequencies were estimated from polymorphic sites in the EBG and RAP populations, averaged across the two, and provided as input to BCFtools. VCF files included in the analysis were those from GATK (McKenna et al. 2010).

Candidate LOH regions were defined as contiguous tracts longer than 1,000 bp with high-confidence HMM scores. The LOH rate (*μ*) for each MA line was calculated using the formula: *μ* = *x*/(*g* × *n*), where *x* is the number of LOH sites observed, *g* is the number of MA generations, and *n* is the number of heterozygous sites in the ancestral genotype. The minimum span of each LOH region was defined as the distance between the first and last sites transitioning from heterozygous to homozygous. The maximum span was defined by including the flanking heterozygous positions.

To distinguish LOH caused by gene conversion from that caused by heterozygous deletions, we examined local sequencing depth. For each MA line, site-specific read depth was standardized by dividing coverage at each site by the mean coverage across all callable sites in the same line. An LOH tract was classified as a putative heterozygous deletion if standardized coverage at the affected region was ≤70% of both the flanking regions and the genome-wide average, while the same region in other lines showed ≥80% standardized coverage relative to the same benchmarks. LOH regions that did not meet these criteria and exhibited coverage comparable to surrounding regions were interpreted as gene conversion events.

## Supplementary Material

msag037_Supplementary_Data

## Data Availability

The FASTQ files of the raw sequencing data for the *D. obtusa* MA lines are available in the NCBI Sequence Read Archive under accession number PRJNA1272726, and the population sequencing data are available under accession number SAMN18588095 (RAP) and SAMN18588093 (EBG). The *D. obtusa* genome assembly is available in NCBI GenBank under accession number PRJNA1272018 and the corresponding annotation can be found at https://osf.io/a9pf3/files/osfstorage. The Illumina RNA-seq reads and full-length Iso-Seq reads used for genome annotation have been deposited in the NCBI Sequence Read Archive (SRA) under accession ID SAMN52923531. The genome assembly of *D. pulex* (KAP4) and its annotation are available at NCBI under accession number GCF_021134715.1.
