# Supplementary material for: The mutation landscape of Daphnia obtusa reveals evolutionary forces shaping genome stability
Source: Mol Biol Evol. 2026 Feb 9;43(2):msag037. doi: 10.1093/molbev/msag037 (PMC12926508; doi:10.1093/molbev/msag037)

**Table S1.** Summary of repetitive elements in the *D. obtusa* genome annotated by RepeatMasker. At the subfamily level, *Unclassified elements* belong to a known repeat type but cannot be classified into any known subclass. Causes include species-specific or highly diverged elements, fragmented copies, and incomplete database coverage. At the major class level, *Unclassified elements* include repeats that cannot be confidently assigned to any known repeat type.

| **Class** | **Subclass** | **Elements (count)** | **Length (bp)** | **% of Genome** |
| --- | --- | --- | --- | --- |
| Retrotransposons |  | 8,264 | 4,957,321 | 3.83 |
|  | SINEs | 1,099 | 492,323 | 0.38 |
|  | LINEs | 1,035 | 696,173 | 0.54 |
|  | L2/CR1/Rex | 245 | 97,292 | 0.08 |
|  | R1/LOA/Jockey | 47 | 41,797 | 0.03 |
|  | R2/R4/NeSL | 77 | 109,590 | 0.08 |
|  | L1/CIN4 | 418 | 91,968 | 0.07 |
|  | Unclassified | 248 | 355,526 | 0.28 |
|  | LTR elements | 6,130 | 3,768,825 | 2.91 |
|  | BEL/Pao | 1,666 | 1,238,870 | 0.96 |
|  | Ty1/Copia | 419 | 190,100 | 0.15 |
|  | Gypsy/DIRS1 | 3,165 | 2,013,736 | 1.56 |
|  | Retroviral | 255 | 94,996 | 0.07 |
|  | Unclassified | 625 | 231,123 | 0.17 |
| DNA transposons |  | 3,539 | 1,252,693 | 0.97 |
|  | hobo-Activator | 1,054 | 365,664 | 0.28 |
|  | Tc1-IS630-Pogo | 62 | 32,615 | 0.03 |
|  | PiggyBac | 284 | 156,598 | 0.12 |
|  | Tourist/Harbinger | 447 | 148,123 | 0.11 |
|  | Other (Mirage / P-element / Transib) | 69 | 20,238 | 0.02 |
|  | Unclassified | 1,623 | 529,455 | 0.41 |
| Rolling-circles |  | 142 | 139,907 | 0.11 |
| Unclassified |  | 63,242 | 20,904,327 | 16.16 |
| Small RNA |  | 100 | 90,946 | 0.07 |
| Satellites |  | 323 | 207,524 | 0.16 |
| Simple repeats |  | 61,700 | 2,647,182 | 2.05 |
| Low complexity |  | 11,112 | 498,241 | 0.39 |
| Total repeats | (All categories combined) |  | 30,698,141 | 23.73 |

**Table S2.** Comparison of genome assembly and annotation statistics between *D. obtusa* and *D. pulex*. PCGs, protein-coding genes; lncRNAs, long non-coding RNAs.

|  | *D. obtusa* | | | | *D. pulex* | | | |
| --- | --- | --- | --- | --- | --- | --- | --- | --- |
| Chr. | Size (Mb) | # Contig | PCGs | lncRNA | Size (Mb) | # Contig | PCGs | lncRNA |
| Chr.1 | 7.1 | 12 | 871 | 266 | 8.3 | 1 | 1,029 | 200 |
| Chr.2 | 12.9 | 32 | 1,328 | 455 | 13.4 | 2 | 1,325 | 363 |
| Chr.3 | 11.6 | 24 | 1,293 | 453 | 13.3 | 1 | 1,386 | 355 |
| Chr.4 | 9.6 | 19 | 1,002 | 371 | 9.2 | 2 | 969 | 255 |
| Chr.5 | 10.6 | 25 | 1,208 | 429 | 12.0 | 1 | 1,257 | 408 |
| Chr.6 | 8.3 | 15 | 951 | 345 | 7.3 | 1 | 863 | 226 |
| Chr.7 | 11.5 | 18 | 1,711 | 521 | 12.3 | 1 | 1,657 | 446 |
| Chr.8 | 14.4 | 31 | 1,379 | 450 | 14.2 | 2 | 1,428 | 371 |
| Chr.9 | 10.4 | 20 | 1,202 | 477 | 9.6 | 1 | 1,162 | 410 |
| Chr.10 | 14.8 | 23 | 2,109 | 596 | 16.3 | 1 | 2,101 | 495 |
| Chr.11 | 7.0 | 16 | 1,055 | 376 | 6.6 | 1 | 945 | 270 |
| Chr.12 | 11.0 | 22 | 1,203 | 477 | 10.7 | 2 | 1,160 | 333 |
| Sum | 129.3 | 257 | 15,312 | 5,216 | 133.2 | 17 | 15,282 | 4,132 |

**Table S3.** Mapped reads and coverage for the samples. The number before the hyphen in the sample ID represents the clone, while the number after the hyphen represents the generation number. Breadth of coverage is the number of genome bases covered by at least one read divided by the genome size 129,356,989 bp. Depth of coverage refers to the number of times a nucleotide is covered by sequencing reads.

| **Sample ID** | **Reads** | **Breadth of coverage** | **Depth of coverage** |
| --- | --- | --- | --- |
| 13-507 | 66,601,884 | 84.8% | 79 |
| 14-465 | 114,753,386 | 85.5% | 135 |
| 20-479 | 48,315,826 | 84.3% | 57 |
| 26-460 | 70,892,331 | 84.9% | 84 |
| 27-508 | 72,565,846 | 84.9% | 86 |
| 28-503 | 41,518,114 | 84.0% | 49 |
| 33-427 | 72,007,145 | 84.9% | 85 |
| 48-504 | 46,911,072 | 84.0% | 56 |
| Average | 66,695,701 | 84.7% | 79 |

**Table S4.** Summary of genomic site filtering criteria used in this study: (a) sites with read coverage between 20 and 300; (b) heterozygous sites predicted by GATK; (c) sites passing a binomial test for the expected 1:1 allele ratio; and (d) additional filters, including thresholds for minor allele frequency, strand bias, mapping and read position quality, as well as a hard filter removing sites with fewer than five ALT-supporting reads based on the AD field.

| Sample | 20≤x≤300^a^ | GATK^b^ | Binomial test^c^ | Final^d^ |
| --- | --- | --- | --- | --- |
| 13-507 | 88,622,775 | 508,191 | 429,579 | 425,191 |
| 14-465 | 91,015,198 | 519,821 | 425,002 | 422,045 |
| 20-479 | 84,616,425 | 502,945 | 423,115 | 416,038 |
| 26-460 | 89,212,784 | 509,926 | 430,328 | 426,034 |
| 27-508 | 89,388,973 | 497,307 | 418,662 | 414,541 |
| 28-503 | 82,208,355 | 465,554 | 384,926 | 376,442 |
| 33-427 | 89,290,523 | 514,800 | 434,364 | 430,178 |
| 48-504 | 83,730,680 | 459,081 | 383,013 | 376,073 |
| **Average** | **87,260,714** | **497,203** | **416,124** | **410,818** |

**Table S5.** Nuclear substitution spectra (counts) and transition-to-transversion (Ts:Tv) ratios across eight mutation accumulation (MA) lines of *Daphnia obtusa*.

|  |  |  |  |  |  |  |  |
| --- | --- | --- | --- | --- | --- | --- | --- |
| MALines | CG>TA | AT>GC | CG>GC | CG>AT | AT>CG | AT>TA | Ts:Tv |
| 13-507 | 33 | 26 | 9 | 10 | 14 | 20 | 1.11 |
| 14-465 | 32 | 20 | 11 | 15 | 4 | 16 | 1.13 |
| 20-479 | 76 | 57 | 9 | 39 | 28 | 37 | 1.18 |
| 26-460 | 68 | 45 | 12 | 24 | 13 | 32 | 1.40 |
| 27-508 | 52 | 34 | 16 | 23 | 17 | 22 | 1.10 |
| 28-503 | 69 | 47 | 4 | 17 | 17 | 28 | 1.76 |
| 33-427 | 79 | 45 | 17 | 26 | 17 | 24 | 1.48 |
| 48-504 | 88 | 58 | 11 | 32 | 34 | 36 | 1.29 |
| Total/Mean | 497 | 332 | 89 | 186 | 144 | 215 | 1.31 |

**Table S6**. Summary of variant effects by impact level, as estimated by SnpEff (Cingolani et al. 2012). Each gene may have multiple effects in SnpEff; here, only the effect with the largest impact was used. Estimates from simulations are presented as mean ± SE. Impact categoriesare defined as HIGH (e.g., nonsense), MODERATE (e.g., missense), LOW (e.g., synonymous), and MODIFIER (non-coding variants, e.g., introns, UTRs, or intergenic). The percent from simulation was generated by performing 100 simulations of the same number of SNMs, maintaining the observed transition-to-transversion ratio, and annotated them using snpEff.

| Type | Observed (%) | Simulated (%) |
| --- | --- | --- |
| High | 2.0% | 2.8%±0.10% |
| Moderate | 13.9% | 23.7%±0.26% |
| Low | 8.6% | 9.2%±0.19% |
| Modifier | 75.4% | 64.3%±0.34% |

**Table S7.** summarizes the *D. obtusa* population collected in this study. EBG and RAP are the ones used the check the power of selection in natural clones.

| Population | Long name | State (USA) | Latitude, Longitude | Year | Live clones |
| --- | --- | --- | --- | --- | --- |
| **EBG** | **Edinburgh** | **MO** | **40.0814, -93.6938** | **2015** | **130** |
| PYR | Pyramid Court | IN | 39.2099, -86.5793 | 2014 | 108 |
| AQP |  | AL | 33.0584,-87.6406 | 2016 | 72 |
| JP |  | GA | 33.5658,-85.0872 | 2016 | 9 |
| **RAP** | **Refuge Admin** | **MS** | **33.2691,-88.8088** | **2016** | **119** |
| RZP |  | AR | 33.8089,-92.8897 | 2016 | 46 |
| TRH |  | AL | 33.0584,-87.6404 | 2016 | 116 |

**Table S8.** Summary of SNPs by functional category. *Shared* denotes variants found in both EBG and RAP.

| Region Type | Total | EBG | RAP | Shared |
| --- | --- | --- | --- | --- |
| Exonic | 296 | 64 | 64 | 23 |
| synonymous | 93 | 19 | 19 | 8 |
| nonsynonymous | 203 | 45 | 45 | 15 |
| Intronic | 323 | 95 | 110 | 46 |
| Intergenic | 548 | 178 | 219 | 114 |

**Table S9.** Summary of gene conversion level events across MA lines.
The conversion rate for each MA line was calculated using the formula: µ = *x*/(*g* × *n*), where *x* is the number of conversion sites observed, *g* is the number of MA generations, and *n* is the number of heterozygous sites in the ancestral genotype. Mean conversion tract lengths and the number of sites per event are shown with their corresponding standard errors in parentheses. *Total* indicates the number summarized across all lineages.

| **Sample ID** | **Events** | **Mean Length (kb)** | **Mean Sites** | **Conversion Rates** |
| --- | --- | --- | --- | --- |
| 13-507 | 0 | NA | NA | NA |
| 14-465 | 0 | NA | NA | NA |
| 20-479 | 3 | 14 (7) | 60 (53) | 8.36×10-6 |
| 26-460 | 6 | 404(205) | 578 (226) | 2.40×10-5 |
| 27-508 | 1 | 412 (NA) | 1595 (NA) | 1.02×10-5 |
| 28-503 | 7 | 452(343) | 1136 (745) | 5.60×10-5 |
| 33-427 | 5 | 385(34) | 164 (140) | 6.05×10-6 |
| 48-504 | 18 | 363(102) | 935 (284) | 1.36×10-4 |
| Total | 40 | 14,762 | 30,868 | 2.62×10^-5*^ |

**Figure S1. Synteny map between *Daphnia obtusa* and *Daphnia pulex*.**
The map was generated by comparing the whole-genome assemblies of *D. obtusa* and *D. pulex* using the online tool [D-GENIES](https://dgenies.toulouse.inra.fr).


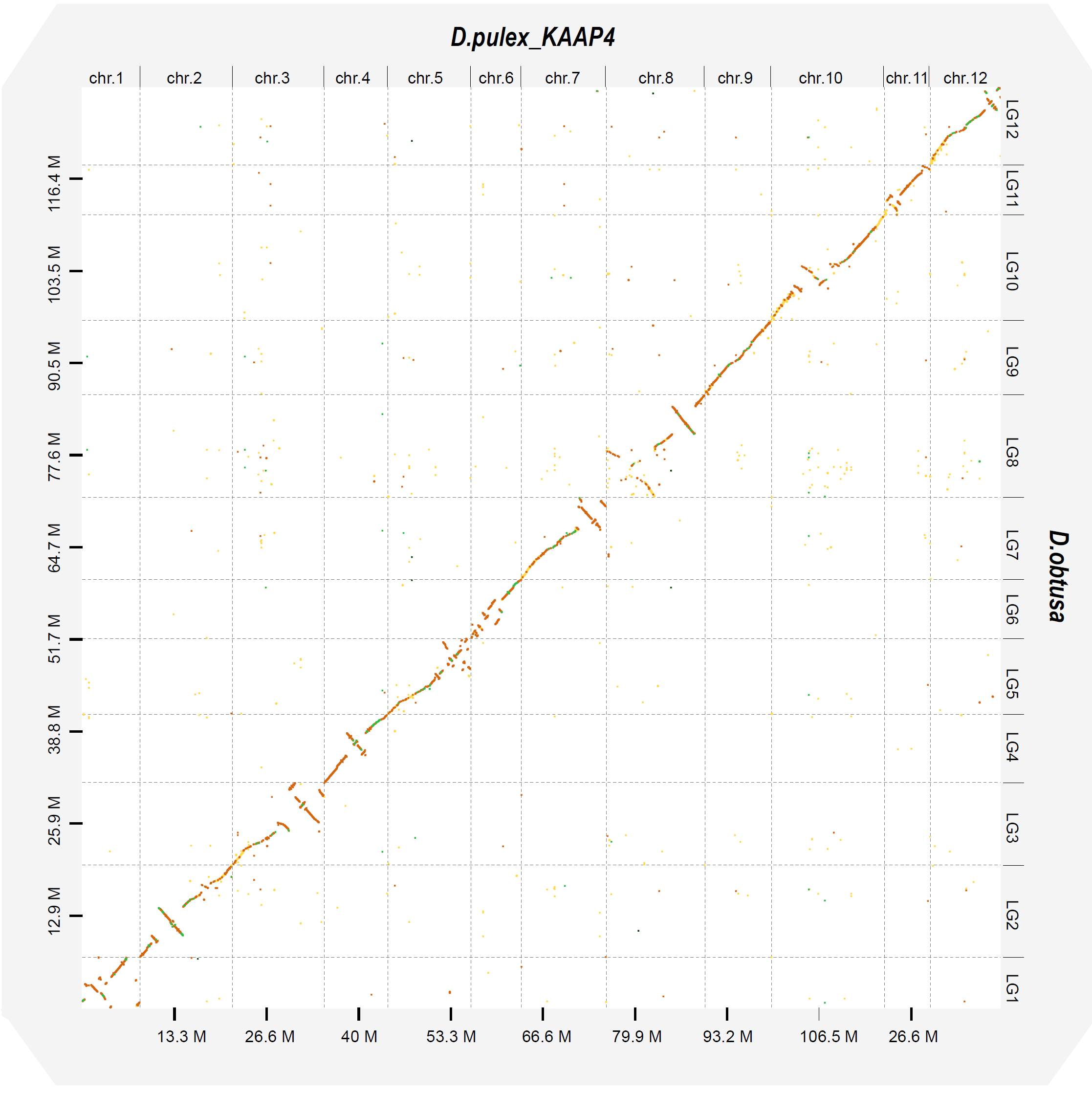


**Figure S2**. Comparison of indel mutation rates among intergenic, intronic, and exonic regions.


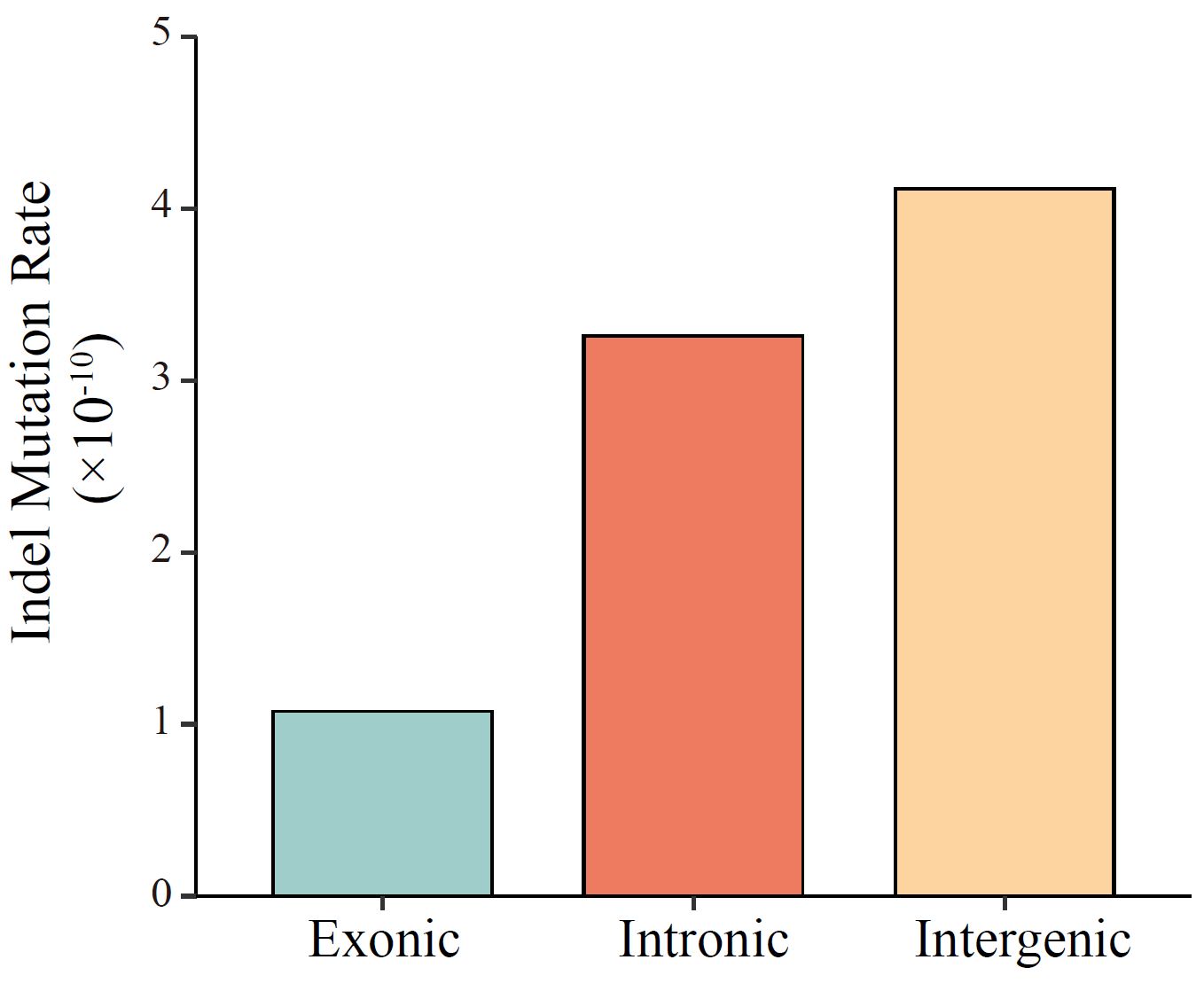


**Figure S3**. Mutation rates across the 12 chromosomes reveal no statistically significant differences.

**
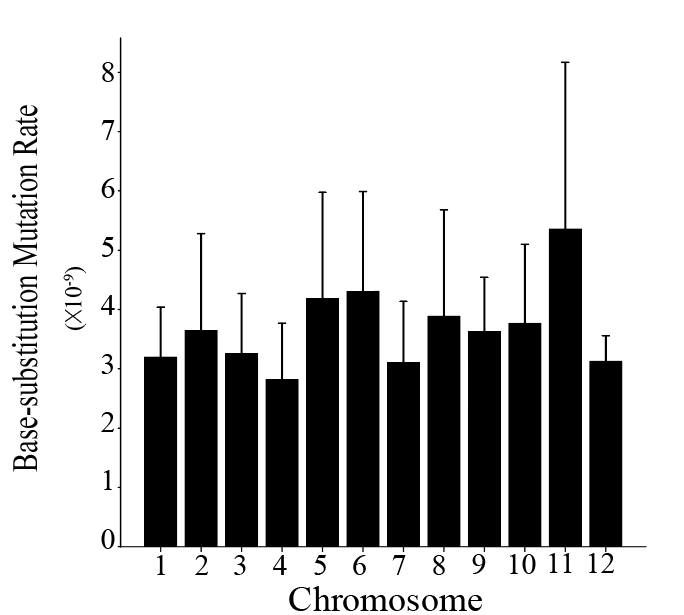
**

**Figure S4**. Relationship between gene expression level and deviation from neutral GC expectation in *D. obtusa*. Scatter plot showing the relationship between ΔGC (the deviation of GC content in the coding sequence of each gene from the neutral expectation of 0.32; X-axis) and gene expression level (normalized to TPM; Y-axis). Each point represents the mean expression level of genes within a given ΔGC bin.


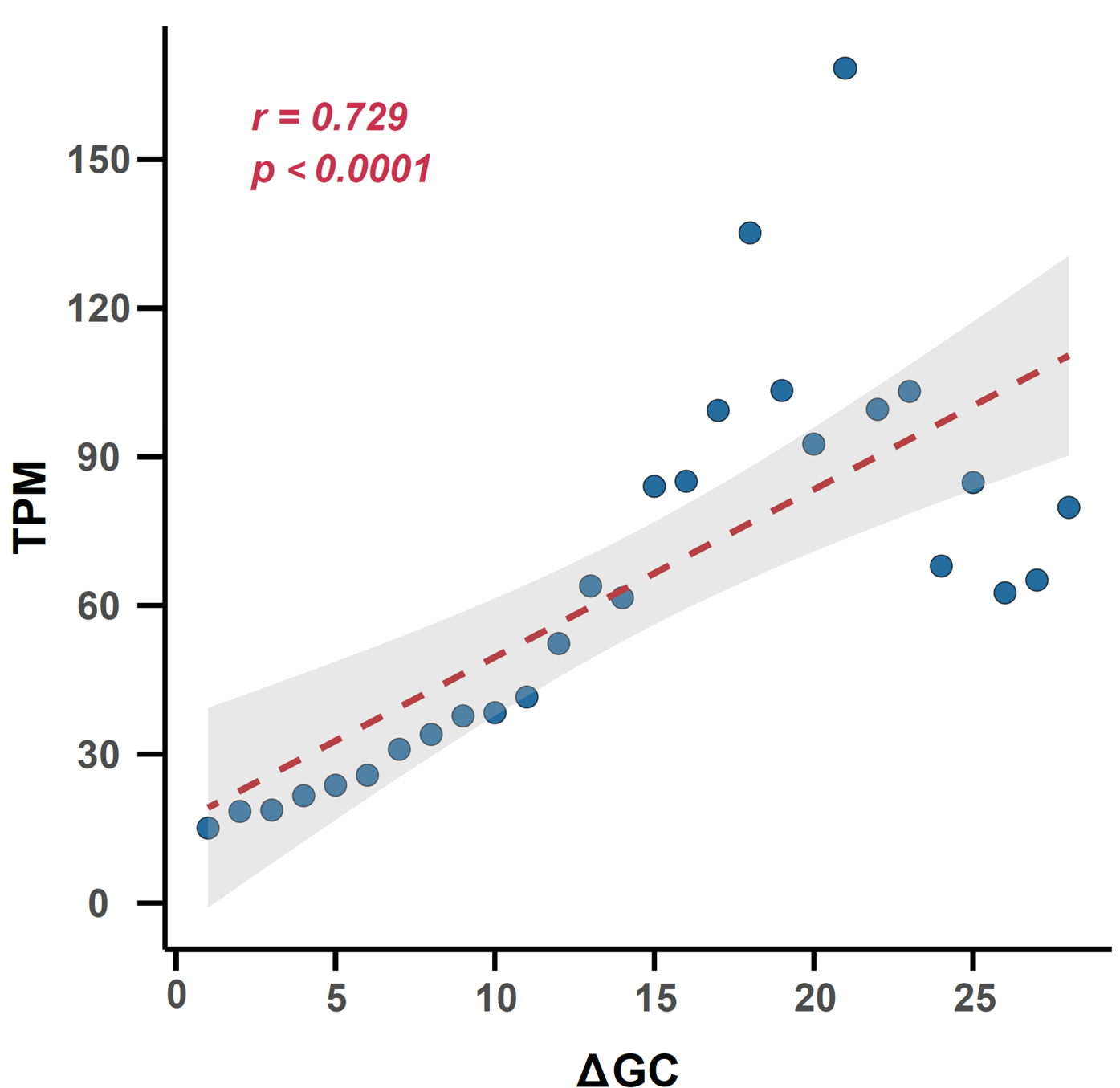


**Figure S5.** Comparison of gene expression levels between mutated and non-mutated genes. The median expression depth (in transcripts per million, TPM) was compared between the two categories.


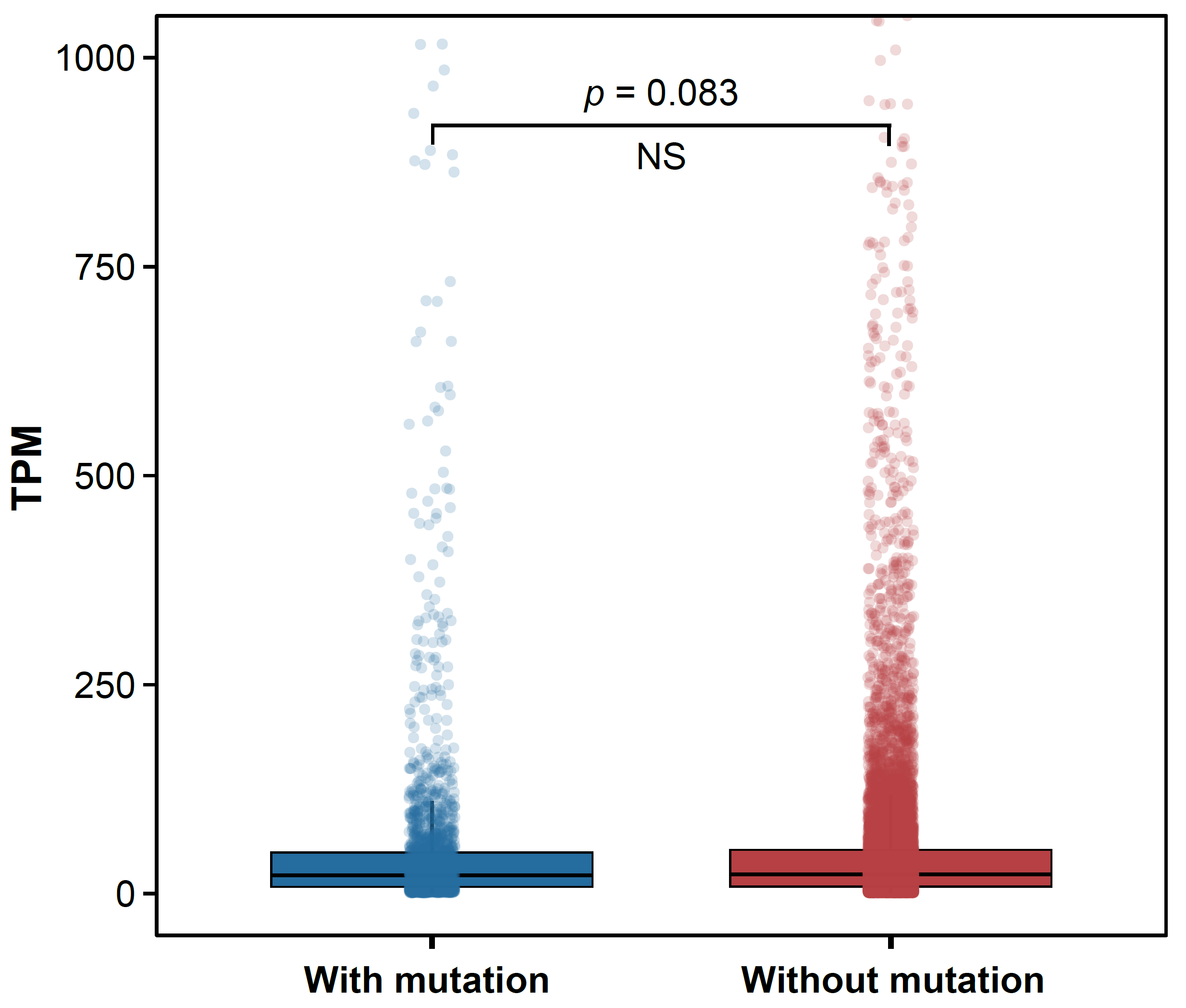

Supplement: msag037_Supplementary_Data [file msag037_supplementary_data.zip › Supplementary Tables and Figures.docx]
